# Study of mentalizing ability in borderline personality disorder: relationship with impulsivity

**DOI:** 10.1192/j.eurpsy.2023.310

**Published:** 2023-07-19

**Authors:** A. Galvez-Merlin, P. de la Higuera-Gonzalez, J. M. Lopez-Villatoro, A. de la Torre-Luque, M. Diaz-Marsa, J. L. Carrasco

**Affiliations:** 1 Department of Legal Medicine, Psychiatry and Pathology, Faculty of Medicine, Universidad Complutense de Madrid; 2 Health Research Institute, Hospital Clínico San Carlos (IdISSC); 3 Department of Personality, Assessment and Clinical Psychology, Universidad Complutense de Madrid; 4 Biomedical Research Networking Consortium for Mental Health (CIBERSAM), Madrid, Spain

## Abstract

**Introduction:**

Borderline personality disorder (BPD) is a severe mental disorder characterized by affective, behavioral and relational instability, along with interpersonal hypersensitivity and unstable affective relationships (APA 2013). Poor interpersonal functioning could be associated with critical deficits in the ability to mentalize in these patients, together with high levels of impulsivity. Although most studies have described hypermentalization deficits among BPD patients (Bora Psychol Med 2021;51 2541-2551), existing literature is still scarce on this aspect, as well as its relationship with the impulsive behavior.

**Objectives:**

1) to assess specific mentalizing deficits in BPD compared to healthy controls in a complex ecological mentalization task; 2) evaluate the relationship between mentalization and impulsivity in BPD.

**Methods:**

63 patients diagnosed with borderline personality disorder and 31 control subjects were studied using the Movie for the Assessment of Social Cognition -MASC- (Dziobeck et al. J Autism Dev Disord 2006; 36 623-636) and the Barratt Impulsivity Scale -BIS-11- (Patton et al. J Clin Psychol 1995; 51 768-774), as well as other sociodemographic and clinical factors. The clinical research study was approved by the Clinical Research Ethics Committee of the Hospital Clínico San Carlos (Madrid, Spain).

**Results:**

The results showed significant differences in the scores related to correct mentalization, hypomentalization, and non-mentalizing responses between patients and controls, with BPD patients showing worse performance. A significant negative relationship was also observed between impulsivity scores and correct mentalizing responses in BPD patients.

**Conclusions:**

The results showed a deficit in the ability to mentalize in BPD patients, compared to control subjects, characterized by a hypomentalization and an absence of mentalization. Likewise, this deficit in mentalization ability was related to greater impulsive behavior in patients. These results would be consistent with the hyperarousal hypothesis in BPD, which would reduce inhibitory control, causing mentalization deficits (Euler et al. J Pers Disord. 2021; 35 177-193). Future studies will try to associate specific impulsive behaviors associated with the characteristics of hypomentalization and absence of mentalization observed in our results.

**Disclosure of Interest:**

None Declared

